# Microblogging the ISMB: A New Approach to Conference Reporting

**DOI:** 10.1371/journal.pcbi.1000263

**Published:** 2009-01-30

**Authors:** Neil Saunders, Pedro Beltrão, Lars Jensen, Daniel Jurczak, Roland Krause, Michael Kuhn, Shirley Wu

**Affiliations:** 1 School of Molecular and Microbial Sciences, University of Queensland, St. Lucia, Brisbane, Queensland, Australia; 2 Department of Cellular and Molecular Pharmacology, University of California San Francisco, San Francisco, California, United States of America; 3 Novo Nordisk Foundation Center for Protein Research, Panum Institute, Copenhagen, Denmark; 4 Department of Bioinformatics, University of Applied Sciences, Hagenberg, Freistadt, Austria; 5 Max-Planck-Institute for Molecular Genetics, Berlin, Germany; 6 European Molecular Biology Laboratory, Heidelberg, Germany; 7 Stanford Medical Informatics, Stanford University, Stanford, California, United States of America; University of California San Diego, United States of America

The International Conference on Intelligent Systems for Molecular Biology (ISMB) has become an important communication hub for bioinformaticians, and the core element of the Conference—presentations of peer-reviewed papers—is now only one of many activities. Presentation of timely journal publications (the Highlights sessions), Special Sessions organized by experts in the respective fields, Tutorials, and Special Interest Group meetings should attract attendees who might otherwise prefer smaller, more focused meetings. In addition to these formal activities, an important aspect is the informal communication between participants. This year, about 1,600 participants attended the meeting in the conference center under the CN Tower in Toronto. ISMB 2008 also left a footprint on the Web, via a Web service named FriendFeed (http://friendfeed.com/), to capture highlights from the Conference in near real time. FriendFeed allows users to share items, either directly or by importing their latest content from any Web site that generates an RSS feed, leading to a continuous stream of information around which communities build (Figure 1). In addition, and of most relevance to ISMB, FriendFeed acts as a microblogging platform: Users post short, typically single-sentence messages which generate conversations in the ensuing comment threads. Microblogging, best exemplified by the Twitter service (http://twitter.com/), is popular in the IT/tech sector, but little used by life scientists. It may be thought of as the fusion of instant messaging and traditional blogs: Anyone can follow or join a conversation, and conversations are archived. We recommend an article by Cameron Neylon entitled FriendFeed for Scientists: What, Why, and How? (http://blog.openwetware.org/scienceintheopen/2008/06/12/friendfeed-for-scientists-what-why-and-how/) for an introduction.

**Figure 1:**
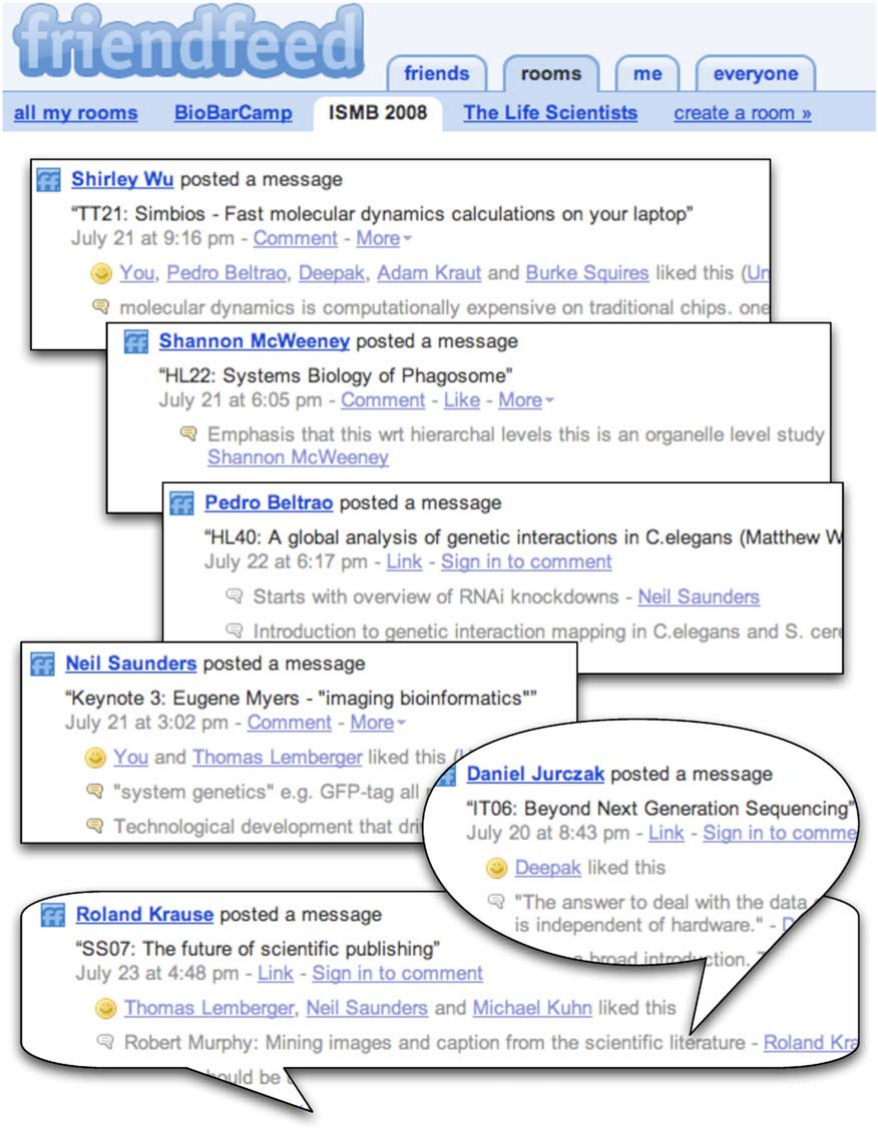
A collage of conversations from the FriendFeed ISMB 2008 room

We—a group of science bloggers, most of whom met in person for the first time at ISMB 2008—found FriendFeed a remarkably useful tool for taking notes and sharing them online. With a core group of ten contributors, we covered parallel sessions, leading to a more comprehensive set of notes than a single person could take. Some presentations, particularly the Keynotes, were covered by several people, generating a detailed overview of a talk from different perspectives. A virtual meeting space—a “room”—was created containing material relevant to ISMB 2008, in which members posted an item detailing the upcoming presentation. Notes on the presentation then took the form of comments posted by the attendees. This proved effective: In addition to notes, questions were asked and answered, links to relevant resources were posted, and comments were even added by people not attending the Conference.

In addition to the live information stream, the ISMB 2008 room is a permanent, searchable archive from which this meeting report was compiled. It is freely available on the Web at http://friendfeed.com/rooms/ismb-2008/.

## Keynotes

The eight Keynote talks this year were noted for their high quality and breadth and were covered online with long comments threads.

Claire Fraser-Liggett opened the meeting with a review of metagenomics and an introduction to the human microbiome project (http://friendfeed.com/search?q=room%3Aismb‐2008+microbiome+OR+fraser). The subsequent Q&A session covered many of the exciting challenges for those working in this field. Clearly, solutions to problems that we thought were largely solved from research into single genomes, such as assembly and genome annotation, are being redefined in the context of metagenomics. What better way to start the meeting than to define the open questions and to highlight the opportunities for bioinformaticians to contribute to the field.

Day one closed with David Jaffe's excellent Introduction to Next-Generation Sequencing (http://friendfeed.com/e/685a365c-9ef6-4ec1-b5b7-48e2619a0790/Keynote-2-enormous-amounts-of-sequencing/). David illustrated the technology using a range of examples: chromatin modification, high-throughput polymorphism discovery in bacterial and eukaryotic genomes, and, finally, de novo genome assembly using short reads. He introduced some of the new tools required to work with short reads, such as the ALLPATHS assembly algorithm, demonstrating again how new technology drives software development in bioinformatics. Next-generation sequencing continues to gain attention, and David's enthusiasm for the topic led one commenter to note that “short reads sequencing can be used as THE general-purpose tool.”

Eugene Myers is a regular guest at ISMB. In light of his major contributions to sequence analysis, it may surprise you to learn that his new passion is Image Analysis (http://friendfeed.com/e/60f95dea-7f5d-454d-a296-abddbeb69cab/Keynote-3-Eugene-Myers-imaging/). The take-home message from Eugene's Keynote on day two was that digital microscopy is now a high-throughput technique, with the potential to generate terabytes of data. Noting that “my life is very colorful these days,” he presented a diverse set of analyses: tubulin tracking in mitosis, stereotypy-tracking cell position during worm development, motion tracking of mouse whiskers, and, finally, early results from ambitious projects to create atlases of fly and mouse brain. His “worm straightening algorithm” was particularly well-received by the audience. Noting that image analysis focuses currently on modeling and annotation, Eugene outlined the ultimate goal: the ability to mine information from this type of experimental data.

The second Keynote of day two was a highlight: the presentation of the Overton Prize to Aviv Regev. In her presentation Modular Biology: The Function and Evolution of Molecular Networks (http://friendfeed.com/e/29b67aac-38f2-4dd5-8c19-b8596895d36e/Keynote-Aviv-Regev-on-Modular-Biology/), Aviv looked beyond the traditional representation of biological networks as, to quote a FriendFeed comment, “nasty hairball diagrams,” to ask: Is the concept of a functional module useful? Judging by her research, the answer is a resounding “yes.” Aviv showed the application of the modular concept to biological systems including evolution of the regulation of gene expression and analysis of gene expression in cancer. Unusual for keynotes, she also presented novel data: a comparative metabolic analysis of carbon utilization across 13 yeast species. The evolution of networks was a key theme and linked well to the final Keynote of the Conference. Aviv concluded that the temporal and spatial key variables are still missing from network models.

The Keynote presentations took a more clinical turn on day three, with Morag Park on Profiling the Breast Tumor Microenvironment (http://friendfeed.com/e/04150591-adf3-4e16-a6ed-7fbeaf7d5ac9/Keynote-5-Morag-Park-profiling-the-breast-tumor/). Proving that the ISMB is as much about biologically relevant research findings as computation and algorithm development, Morag illustrated how new microdissection techniques enable gene expression analysis in subpopulations of tumor cells. Having identified distinct expression profiles for different cell types, her research team has developed predictors that correlate with clinical outcomes and permit customization of individual treatment. Her presentation was an excellent demonstration that bioinformatics can impact directly the quality of human life.

In the afternoon session, we witnessed a tour-de-force Overview of Systems Biology (http://friendfeed.com/e/ef87914f-c3c5-4943-88ad-c518e9991fb4/Keynote-6-Bernhard-Palsson-systems-biology-an-era/) from Bernhard Palsson. His talk led us through a brief history of systems biology to his new paradigm: a comprehensive, structured knowledge base that we can use for metabolic reconstruction. Bernhard stressed the role of community annotation in “filling in the gaps” and described how this works, in the form of annotation jamborees targeted at model organisms. His presentation received the highest number of comments and was clearly of great interest to non-attendees following the coverage.

Small non-coding RNAs continue to be a hot topic. A new twist in the tale was provided by Hanah Margalit in the opening talk of day four on Intriguing Roles for Small ncRNAs in Cellular Regulatory Networks (http://friendfeed.com/e/29ec0f3d-5a1c-4f2d-807a-5f42e959bf50/Keynote-7-Hana-Margalit-intriguing-roles-for/). In another example of the two-way traffic between computational analysis and biological discovery, Hanah's group developed new algorithms to predict ncRNAs and their targets. Applying these algorithms, they demonstrated that viruses use miRNAs to downregulate the host immune system. With infectious enthusiasm, Hanah presented a set of recent experiments that validate this exciting new discovery.

The last of the Keynotes was David Haussler on 100 Million Years of Evolutionary History of the Human Genome (http://friendfeed.com/e/d6af6373-897d-483d-975e-5204ef127f96/Keynote-8-David-Haussler-100-million-years-of/). Improvising effortlessly through a brief technical glitch, he reminded us just how much new biology can be learned by obtaining and comparing complete genome sequences. Examples ranged from the more familiar: ultra-conserved regions in the human genome, the HAR1 gene, which looks increasingly to be a key factor in human brain development, to the almost-quirky—the discovery of an ancient transposon most similar to coelacanth. David concluded with an outline of his grand challenge: reconstructing the evolutionary history of each base in the genome of humans and other mammals. Once again, it was clear that many opportunities exist for bioinformaticians to contribute the tools and analyses required for this task.

## Summary of the Sessions

**Figure 2:**
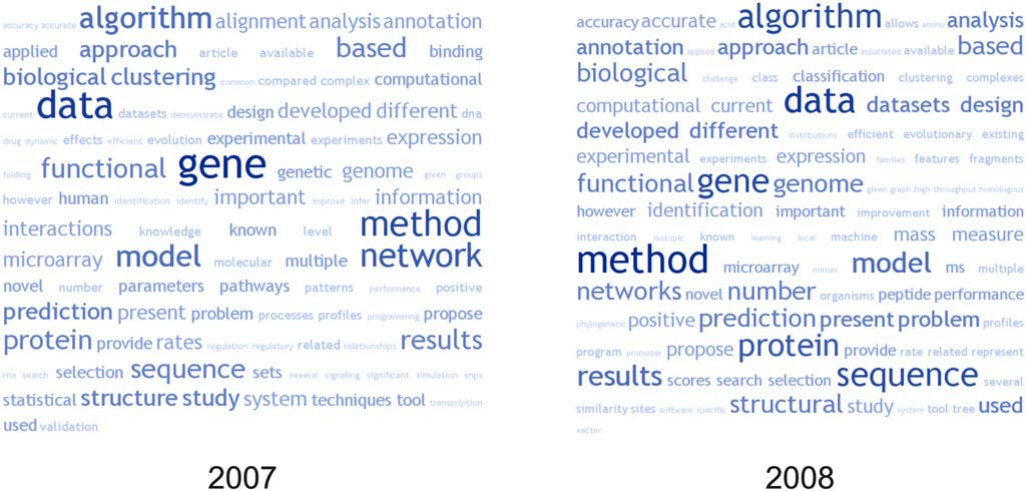
Word cloud analysis of the Proceedings abstracts from ISMB/ECCB 2007 (left) and ISMB 2008 (right)

Our notes are a partial record of ISMB 2008, reflecting the relatively small number of bloggers and their interests. One can imagine that with more contributors, an impression of the most popular presentations and topics could be gained from the level of online coverage. Sessions from the Biopathways SIG, Highlights, Proceedings, and Special Sessions tracks were covered online, in particular those discussing protein–protein interactions, biological networks, and the future of scientific publishing. One approach to provide an overview of the meeting is a “word cloud,” indicating commonly used words mined from Conference Abstracts. Figure 2 shows the 100 most frequently used words from the 2008 ISMB Proceedings Abstracts, compared with the Proceedings from the 2007 ISMB/ECCB meeting in Vienna. These figures were prepared using the Web application TagCrowd (http://tagcrowd.com/).

Although this is a crude analysis, several interesting trends can be discerned. The terms *mass*, *peptide*, and *ms* are on the rise, indicating increased use of mass spectrometry. In 2008, *vector* and *machine* appear, highlighting the current popularity of SVMs in bioinformatics. We also see increased usage of the terms *accuracy/accurate* and *performance*. Although Phil Bourne joked during his presentation on Pharmaceutical Off-Targets (http://friendfeed.com/e/d5b274e5-1d34-4108-853e-5974b2a4cccd/HL29-Phil-Bourne-off-targets-for-some-major/) that he was “too old to compare my method to other methods by showing that we're better on a completely different dataset,” this type of statistical validation seems to be used more widely than ever. *Clustering* is less popular in 2008, and increasingly we are referring to *datasets* rather than just *data* and to *networks* rather than a single *network*. We also discern the ever-growing development of *methods* and *algorithms* and more emphasis on the *biological* and *experimental*.

## Biopathways SIG

The Ninth Annual BioPathways (http://www.biopathways.org/) meeting was dedicated to computational methods for synthetic biology and was organized in collaboration with Emergence (http://www.emergence.ethz.ch/), an EU-funded consortium that fosters synthetic biology in Europe. Over two days, biological pathways were discussed under the topics of network reconstruction, database/software development, and network evolution.

The need for standardization and machine-accessible pathway data was apparent. Peter Karp talked about recent developments at the MetaCyc (http://metacyc.org/) and BioCyc (http://biocyc.org/) pathway databases. The number of available reactions and pathways in these databases has increased at an impressive rate due to curation. The nonredundant MetaCyc database now holds 7,144 reactions from 1,138 pathways. Capturing pathway information locked away in published literature could benefit from voluntary curation. This is the objective and the experiment under way at WikiPathways (http://www.wikipathways.org/index.php/WikiPathways).

The availability of biological information in a machine-readable format improves our ability to move data between analytical tools. Jennifer Gardy gave an interesting example in Cerebral (http://www.pathogenomics.ca/cerebral/), a Cytoscape plugin that facilitates pathway layering according to location information. Cerebral was also developed for the comparison of multiple quantitative datasets, such as gene expression data across different conditions.

Novel network analysis methods were described by David Gilbert and Shoshana Wodak. The Gilbert group has been working on a language to specify semiquantitative model rules that can be used for model verification. This could also be used to search pathway model databases for pathways that can implement a desired behavior for engineering purposes. Shoshana Wodak presented a new algorithm to find paths within a metabolic network, given start and end point metabolites.

Acquisition of pathway information relies increasingly on large-scale datasets. Finding functional relevant pathways through analysis of large datasets is sometimes referred to as network reconstruction, a topic covered by Chris Sander, Lars Juhl Jensen, and Rune Linding. Lars presented NetPhorest (http://netphorest.info/), a compendium of classifiers for linear motifs important for phosphorylation-dependent signaling. Rune described NetworKIN (http://networkin.info/search.php), a tool to predict in vivo kinase substrates by integrating gene functional association scores from the STRING (http://string.embl.de/) database, in vivo phosphorylation data, and classifiers for kinase-substrate recognition obtained from NetPhorest. They explained how integration of the growing list of classifiers from NetPhorest might be used to predict signaling pathways.

Chris Sander challenged the oft-used phrase: “There is no such thing as pathway reconstruction, nor is there such [a] thing as pathway re-engineering. Pathways are simply biological models.” He described an interesting approach to derive biological models from a significant collection of observations obtained from combinatorial perturbation experiments. Using a cancer cell line, researchers performed a set of 21 drug pair treatments and observed phosphoproteins and cell cycle markers. Using a novel computational approach, they were able to rediscover many of the functional interactions among the genes and to highlight potential new connections.

Are pathway models obtained from the literature or from novel mining approaches useful for medical or technological applications? Chris Sander, Phil Bourne, and Christopher Myers all touched on this subject from different angles. Myers told us that all biological pathways are “sloppy.” Their analysis of different models shows that individual parameters are typically poorly constrained by collective fits, even when abundant observations are available. The focus of experimental design should move away from determining precise reaction parameters to collective behaviors. This also implies that altering precisely the activity of one cellular component for medical applications might not be the best approach to follow. Both Chris Sander, in relation to cancer therapy, and Phil Bourne, in relation to prediction of drug off-target effects, mentioned the need to understand the effect of pharmacological interventions within the context of complete biological pathways.

## Web 2.0 for Science Birds of a Feather Session

Many scientists make little use of public Web 2.0 resources for creativity, sharing, and collaboration. Bloggers Shirley Wu (http://shirleywho.wordpress.com/) and Pedro Beltrão (http://pbeltrao.blogspot.com/) co-chaired a Birds of a Feather (BoF) discussion—Science Blogging and Web 2.0—attended by about 20 people. A lively discussion ensued, examining the reluctance and the enthusiasm of life scientists to embrace Web 2.0 (http://van.embl.de/cb/web_20_talk_series.shtml).

The first topic was online reference managers: Connotea (http://www.connotea.org/), CiteULike, (http://www.citeulike.org/), and new entrant Labmeeting (http://www.labmeeting.com/signin), which allow tagging and online storage of papers. Why don't more scientists use them? A major reason seems to be the reluctance to share what one is reading with the rest of the world, based on the fear that a reading list is a window into your research. More generally, scientists are hesitant to use the open Web as an incubator for ideas and would rather rely on a tight circle of trusted individuals.

Other issues discussed included uncertainty surrounding intellectual property of Web content, the problem of critical mass for social networking sites, the reliability and expertise of bloggers, and the possibility of domination by shallow collaborations. Pedro stated that the problem is not one of too many superficial connections, but that too few connections are being made. Tools such as Epernicus (http://www.epernicus.com/) could remedy this by connecting, for example, biologists and bioinformaticians working on complementary research.

Many participants had a positive view of online tools such as social networking and blogging. Roland Krause described how a connection made on a social networking site resulted in collaboration and a journal publication. Participants agreed that blogging or joining communities such as FriendFeed, Nature Network (http://network.nature.com/), and OpenWetWare (http://www.openwetware.org/wiki/Main_Page) can result in authorship, job offers, and a vastly increased audience—at least compared with your papers. Consensus from those who participate in science online is that the Web is a great equalizer: Students and post-docs can easily strike up “conversations” with more established scientists, and contributions have less to do with status and more to do with ideas. Web participation also opens your eyes to other perspectives and enables you to learn from people with more expertise than you.

The BoF session ended with a discussion about coverage of ISMB Toronto. About half of the participants knew about the Conference microblogging. Everyone agreed that coverage of ISMB on the Conference Web site could be improved by something similar. The Conference organizers also agree and hope to include live microblogging at ISMB 2009 in Stockholm.

## Poster Sessions

The scale of ISMB is illustrated by the poster sessions: approximately 800 posters, presented over two evenings. How might poster presentation before, during, and after meetings be improved? Shirley Wu dreams of a future (http://friendfeed.com/e/44118735-65e8-0980-64c3-36247dd8dfab/Off-to-ISMB-2008/) without the problems of printing and transport, where rolls of paper in cardboard tubes are replaced by memory sticks and flat-screen displays.

Shouldn't posters be permanently archived? Sharing posters digitally using an image hosting service met with limited success (http://friendfeed.com/e/4e094687-f68e-5c51-f3ce-fac663f18c23/STRING-and-STITCH-known-and-predicted/). A search for Web sites that host posters located the e-posters archive (http://www.eposters.net/). However, this site serves as a PDF repository and lacks the visual and community features expected. Clearly, an opportunity exists for a well-designed service catering to those who wish to archive and share posters on the Web.

Imagine that you were able to host a poster session at any time, with visitors from anywhere in the world. Organizations including Nature Publishing Group and the American Chemical Society are already running virtual poster sessions, meetings, and interactive displays in Second Life (http://secondlife.com/). In the future, might all scientific conferences live on long after the meeting in virtual environments? Those interested should read Jean-Claude Bradley's description (http://usefulchem.blogspot.com/2008/02/acs-island-on-second-life.html) of how this can be achieved.

## Summary

Microblogging platforms and other tools for videos, podcasts, and virtual environments provide an untapped potential for science conferences. Our experiment using FriendFeed to cover ISMB 2008 was educational and surprisingly successful. We found that it enhanced our note-taking skills, allowed us to compile notes from parallel sessions, attracted wider interest from non-attendees, and, in addition to the “live” aspect, generated a permanent archive of the meeting.

ISMB/ECCB 2009 will be held in Stockholm. We look forward to the new developments in Web usage by scientists that are sure to emerge between now and then. We also anticipate new and exciting ways to report from Stockholm as it happens; perhaps the ISMB/ECCB 2009 Web site will look something like this: http://www.bork.embl.de/˜jensen/ismb2008/keynotes.php.html?

